# Cow's milk and hen's egg anaphylaxis: A comprehensive data analysis from the European Anaphylaxis Registry

**DOI:** 10.1002/clt2.12228

**Published:** 2023-03-26

**Authors:** Ewa Cichocka‐Jarosz, Sabine Dölle‐Bierke, Urszula Jedynak‐Wąsowicz, Dominique Sabouraud‐Leclerc, Alice Köhli, Lars Lange, Nikolaos G. Papadopoulos, Jonathan Hourihane, Katja Nemat, Kathrin Scherer Hofmeier, Stephanie Hompes, Hagen Ott, Lucila Lopes de Oliveira, Thomas Spindler, Christian Vogelberg, Margitta Worm

**Affiliations:** ^1^ Department of Paediatrics Pulmonology, Allergy and Dermatology Clinic Jagiellonian University Medical College Krakow Poland; ^2^ Department of Dermatology Venerology and Allergology Charité – Universitätsmedizin Berlin Corporate Member of Freie Universität Berlin Humboldt‐Universität zu Berlin Berlin Institute of Health Berlin Germany; ^3^ University Hospital Centre Reims France; ^4^ Division of Allergology University Children's Hospital Zurich Zürich Switzerland; ^5^ Department of Paediatrics St. Marien‐Hospital Bonn Germany; ^6^ Allergy Department 2nd Paediatric Clinic University of Athens Athens Greece; ^7^ Division of Infection Immunity and Respiratory Medicine University of Manchester Manchester UK; ^8^ Department of Paediatrics University of Medicine and Health Sciences Royal College of Surgeons in Ireland Dublin Ireland; ^9^ Children's Centre Dresden – Friedrichstadt Dresden Germany; ^10^ Allergy Unit Department of Dermatology University of Basel Cantonal Hospital Aarau Aarau Switzerland; ^11^ Department of Paediatrics Altona Children's Hospital Hamburg Germany; ^12^ Division of Paediatric Dermatology and Allergology Children's Hospital Auf der Bult Hannover Germany; ^13^ Department of Paediatrics Federal University of São Paulo – Escola Paulista de Medicina (UNIFESP‐EPM) São Paulo Brazil; ^14^ Allergy Campus Hochgebirgsklinik Davos Davos Switzerland; ^15^ Department of Paediatric Pulmonology and Allergy University Hospital Carl Gustav Carus Technical University of Dresden Dresden Germany

**Keywords:** children, cow's milk anaphylaxis, European Anaphylaxis Registry, hen's egg anaphylaxis

## Abstract

**Background:**

Cow's milk (CM) and hen's egg (HE) are leading triggers of anaphylaxis in early childhood. The aim of this study was to identify clinical phenotypes and therapeutic measures for CM anaphylaxis (CMA) compared to HE anaphylaxis (HEA) in children up to 12 years of age, based on a large pan‐European dataset from the European Anaphylaxis Registry.

**Methods:**

Data from 2007 to 2020 on clinical phenotypes and treatment from 10 European countries, as well as Brazil, were analysed. The two‐step cluster analysis was used to identify the most frequent phenotypes. For each trigger, three clusters were extracted based on sex, age, and existence of symptoms in four vitally important systems.

**Results:**

Altogether 284 children with CMA and 200 children with HEA were identified. They were characterised as male (69% vs. 64%), infants (65% vs. 61%), with a most frequent grade III of Ring&Messmer classification (62% vs. 64%), in CMA versus HEA, respectively. Respiratory symptoms occurred more often in CMA (91% vs. 83%, *p* = 0.010), especially in infants (89% vs. 79%, *p* = 0.008). Cardiovascular symptoms were less frequent in CMA (30% vs. 44%, *p* = 0.002), in both infants (33% vs. 46%, *p* = 0.027), and older children (25% vs. 42%, *p* = 0.021). The clusters extracted in the CMA group were characterised as: (1) mild dermal infants with severe GI (40%), 2. severe dermal (35%), 3. respiratory (25%). While in HEA group: 1. infants with severe GI and/or reduction of alertness (40%), (2) conjunctival (16%), (3) mild GI without conjunctivitis (44%). The severity of the reaction was independent from the amount of ingested allergen protein, regardless of trigger. The first‐line adrenaline application differed between the countries (0%–92%, as well as the reasons for not administering adrenaline, *p* < 0.001).

**Conclusions:**

Despite the similarity of their age, sex, and severity grade, the clinical profiles differed between the CMA and HEA children. Adrenaline was underused, and its administration was country dependent. Further studies are needed to assess to what extent the differences in the clinical profiles are related to matrix and/or absorption effects, and/or the allergen itself.

## 1 INTRODUCTION

The European Anaphylaxis Registry (EAR) maintains a large online database that allows for comprehensive analysis of anaphylaxis causes, their clinical patterns, and intervention modalities.^1^ Food allergens are the most frequent triggers of anaphylaxis in children.^1^, ^2^, ^3^ Cow's milk (CM) and hen's egg (HE) are the top two triggers during the first 2 years of life (1.3% for CM compared to 0.8% for HE).^4^, ^5^, ^6^, ^7^ Though their prevalence is not very dynamic, the problem is still clinically relevant, even when most infants outgrow both of these food allergies.^8^, ^9^ Nevertheless, for children in whom the symptoms persist there is still a high risk of severe anaphylaxis.^10^ Earlier data from the EAR database on food allergens, such as peanut‐induced anaphylaxis in children and teenagers, or wheat‐induced anaphylaxis in adults, suggest the impact of a given eliciting food on the anaphylaxis phenotype.^11^, ^12^

These data, and the fact that there are no studies directly evaluating the differences between cow's milk anaphylaxis (CMA) and hen's egg anaphylaxis (HEA) symptomatology, prompted us to perform a comprehensive analysis of CMA and HEA in children, to obtain new insights into their clinical profiles and treatment strategies.

## 2 METHODS

The Online Anaphylaxis Registry ANAPHYLAXIE.net was created to collect data on severe allergic reactions by means of an online questionnaire. The initiative was addressed to physicians with a request to centrally report anaphylactic reactions in their clinical practice. The original version of the questionnaire was developed in Germany for German‐speaking countries. Subsequently, other language versions were created by the authors of original version, and formed the basis for the European Anaphylaxis Registry (EAR).^1^ There is no free access to the database. Researchers, within centres affiliated in the registry, receive an access code that allows them to enter data.

Data used in the current analysis come from tertiary referral centres specializing in paediatric allergology, dermatology, or both, in Germany, France, Austria, Switzerland, Spain, Greece, Italy, Ireland, Bulgaria, Poland, and Brazil (Figure S1). Professionals at the referral centres were required to enter the patients' details into the registry within 1 year of their anaphylaxis episode. The study was registered at ClinicalTrials.gov (Identifier: NCT05210543), and it was approved by both the ethics committee at the Charité ‐ Universitätsmedizin Berlin, in Germany (EA1/079/06), as well as the ethics committees local to the participating referral centres.

### 2.1 Study population

For the purpose of this study, the data of children up to 12 years old who experienced Ring and Messmer (R&M) grade II‐IV CMA and HEA, were extracted from the Registry. According to the original R&M classification, grade II affects at least two organs or systems; grade III means signs of circulatory and/or respiratory failure or shock, and grade IV is circulatory or respiratory arrest, up to fatality. The inclusion and exclusion criteria for this analysis are summarized in supplementary Figure 2 (Figure S2).

The parents or caregivers of the patients signed voluntary informed consent forms for participation in the questionnaire study, which covered the history and details of their child's anaphylaxis episodes. Subsequently, anonymized retrospective results of their diagnosis and counselling were introduced into the Register by specialist medical staff at the participating centres. Only at the centre, the patient originated from, it was possible to identify any sensitive patient's data.

### 2.2 Questionnaire versions and data collected

Data was collected by means of yearly updated questionnaires, versioned from 2 to 8 (for version 2 *n* = 12, for version 3 *n* = 18, for version 4 *n* = 14, for version 5 *n* = 73, for version 6 *n* = 181, for version 7 *n* = 119 and for version 8 *n* = 67) with entry dates from July 2007 to October 2020. The last version of the survey included in this study, consists of 98 questions, divided into six sections. Questions aim to acquire information on the reaction details (symptoms ‐ clinical presentation from skin, gastrointestinal, respiratory, cardiovascular and other organs; time, biphasic, fatality, location, previous reaction), diagnostic testing (auxological parameters, allergy diagnosis), details of exposure both to the eliciting and exacerbating factors, comorbidities, treatment (who performed the intervention, kind of drugs, possible hospital admission), preventive measures (counselling, prescription of emergency drugs; type, number, and dose of adrenaline autoinjector (AAI)). The response time to the questionnaire is approximately 20 min.

Since information about the food intake was categorized (possible answers were one teaspoon, one tablespoon, one cup, etc.), individual volumes were converted to the corresponding protein content (Figure S3).

The diagnostic certainty of the causative factor has been documented on 2 levels – known versus suspected ‐ based on the individual assessment of the local allergist, without giving reasons for his decision.^1^

### 2.3 Statistical analysis

The analysis was made using the IBM SPSS Statistics 27 for Windows statistical package. Qualitative data was presented as counts and percentages. Quantitative data was shown as medians and interquartile ranges.

Comparisons of qualitative variables were made using the Pearson *χ* ^2^ test, when expected frequencies in more than 80% of cells were higher than 5; in other cases the Fisher exact test was used for the 2 × 2 tables, and the Fisher‐Freeman‐Halton test for all others. Comparisons of quantitative variables between groups were made using the Kruskal–Wallis test.

Cluster analysis is a statistical method which lets for identifying the patterns in the data, which differentiate groups of cases. The two‐step cluster analysis was used to identify the most frequent phenotypes. For each trigger, three clusters were extracted based on sex, age, and existence of symptoms in four vitally important systems.

Separate assessment of factors responsible for the administration of adrenaline was made using a logistic regression model for both CMA and HEA. Potential predictors included: country (due to the low number of cases, Austria, Bulgaria, Italy, and Spain were aggregated into one category); sex, age, previous reaction to the trigger, knowledge of the allergy to the trigger; existence of individual symptoms from four vitally important systems, and the time to onset of the symptoms. In the final models, only the variables that significantly influenced adrenaline administration in at least one trigger were retained.

A *p* value of less than 0.05 was considered significant.

## 3 RESULTS

### 3.1 Cow's milk and hen's egg anaphylaxis in the anaphylaxis registry

In total, 284 episodes of CMA and 200 episodes of HEA were reported among 4234 moderate to severe real‐life (i.e. not challenge‐provoked), food‐induced anaphylactic reactions (Figure S2). Mean age in both trigger groups equalled 2.2 years. Both groups were similar in terms of predominance of children up to 1 year old (65% CMA compared to 61% HEA), male children (69% CMA compared to 64% HEA), with the grade III in the R&M classification, being the most common severity grade of clinical symptoms (62% CMA compared to 64% HEA) (Table 1A). They differed in asthma prevalence (19% CMA compared to 9% HEA), and history of previous reactions to the same allergen (52% CMA compared to 36% HEA) (Table 1A). In comparison to the HEA sufferers, 1.5 times more children with CMA were already diagnosed with an allergy to the culprit trigger, of which 95% in CMA group, and 88% in HEA group, reacted previously to the same allergen.

**TABLE 1:** Characteristics of CMA and HEA groups with respect to the first and the subsequent reaction.

	A. Characteristics of trigger groups					B. Characteristics of repeated reactors				
	Trigger					Trigger				
	CMA (*n* = 284)		HEA (*n* = 200)		*p*	CMA (*n* = 143)		HEA (*n* = 69)		*p*
	*N*	%	*N*	%		*N*	%	*N*	%.	
Sex										
F	89	31	72	36	0.284	43	30	25	36	0.368
M	195	69	128	64		100	70	44	64	
Age					0.166					0.138
Up to 1 year	186	65	122	61	NS	64	45	21	30	**<0.05**
From 2 to 3 years	40	14	40	20	NS	35	24	26	38	**<0.05**
From 4 to 6 years	31	11	26	13	NS	25	18	14	20	NS
More than 6 years	27	10	12	6	NS	19	13	8	12	NS
Skin symptoms	275	98	190	96	0.219	135	96	63	91	0.214
GI symptoms	137	50	102	51	0.818	64	47	35	51	0.620
Respiratory symptoms	257	91	166	83	**0.010**	134	94	58	84	**0.014**
Cardiovascular symptoms	83	30	87	44	**0.002**	40	29	29	43	**0.002**
Ring and Messmer severity grade					0.055					0.372
Grade II	101	35	71	36	NS	57	40	26	38	NS
Grade III	175	62	129	64	NS	82	57	43	62	NS
Grade IV	8	3	0	0	NS	4	3	0	0	NS
Time to onset of symptoms					**p < 0.001**					0.140
Up to 10 min	159	67	90	53	**<0.05**	76	60	31	53	NS
11–30 min	62	26	38	23	NS	37	29	15	25	NS
Over 30 min	16	7	41	24	**<0.05**	14	11	13	22	**<0.05**
Biphasic reaction	2	1	7	4	**0.038**	1	1	1	2	0.534
Hospital admission	88	48	74	52	0.512	46	45	21	41	0.645
PICU	10	5	3	2	0.138	5	5	0	0	0.171
Cofactors*	28	10	29	14	0.153	18	13	17	25	0.027
Current atopy	167	66	126	68	0.512	90	68	50	81	0.061
Current food allergy	49	23	39	26	0.629	34	29	24	44	0.053
Current asthma	53	19	17	9	**0.002**	37	26	7	11	**0.010**
Current AR	47	17	22	11	0.085	34	24	13	20	0.464
Current AD	114	41	97	50	0.065	51	36	37	56	**0.008**
Current all other diseases	30	14	23	15	0.927	7	5	4	6	0.752
Allergy known	113	45	58	32	**0.005**	105	78	49	74	0.515
Previous reaction to allergen	143	52	69	36	**< 0.001**					
Milder reaction	122	93	49	85	0.062					
More severe reaction	25	19	16	26	0.250					
Diagnostic measures					**0.007**					0.226
No	27	11	16	9	NS	14	10	3	5	NS
Before the registered episode	112	45	58	32	**<0.05**	89	67	42	65	NS
After the registered episode	111	44	109	59	**<0.05**	30	23	20	30	NS

*Note*: Bold values indicate significant statistical differences.

Abbreviations: PICU, Paediatric Intensive Care unit; AD, atopic dermatitis; AR, allergic rhinitis.

*Cofactors (CMA vs. HEA): Physical exercise (20 vs. 20), drug (7 vs. 7), stress (2 vs. 3); cofactors in repeated reactors (CMA vs. HEA): Physical exercise (14 vs. 13), drug (3 vs. 3), stress (2 vs. 2).

Specifically, in terms of these being repeated reactors, the infants were CMA predominant, and the children aged 2–3 years old were HEA predominant (Table 1B). Additionally, in the group of repeated reactors, the profile of atopic comorbidities differed according to trigger: asthma was more than twice as prevalent in the CMA sufferers, while atopic dermatitis (AD) was 1.5 times more frequent in the HEA group (Table 1B).

Children with CMA mainly reacted to one tablespoon or half a cup of the offending product (corresponding to 0.34 and 2.60 g of CM protein, respectively), while those with HEA, reacted mainly to one teaspoon or one tablespoon (corresponding to 0.65 and 1.30 g of HE protein, respectively) (*p* < 0.001; Figure S3). The severity of the reaction did not depend on the amount of ingested allergen protein, regardless of the type of allergen.

### 3.2 Clinical phenotype

**FIGURE 1:**
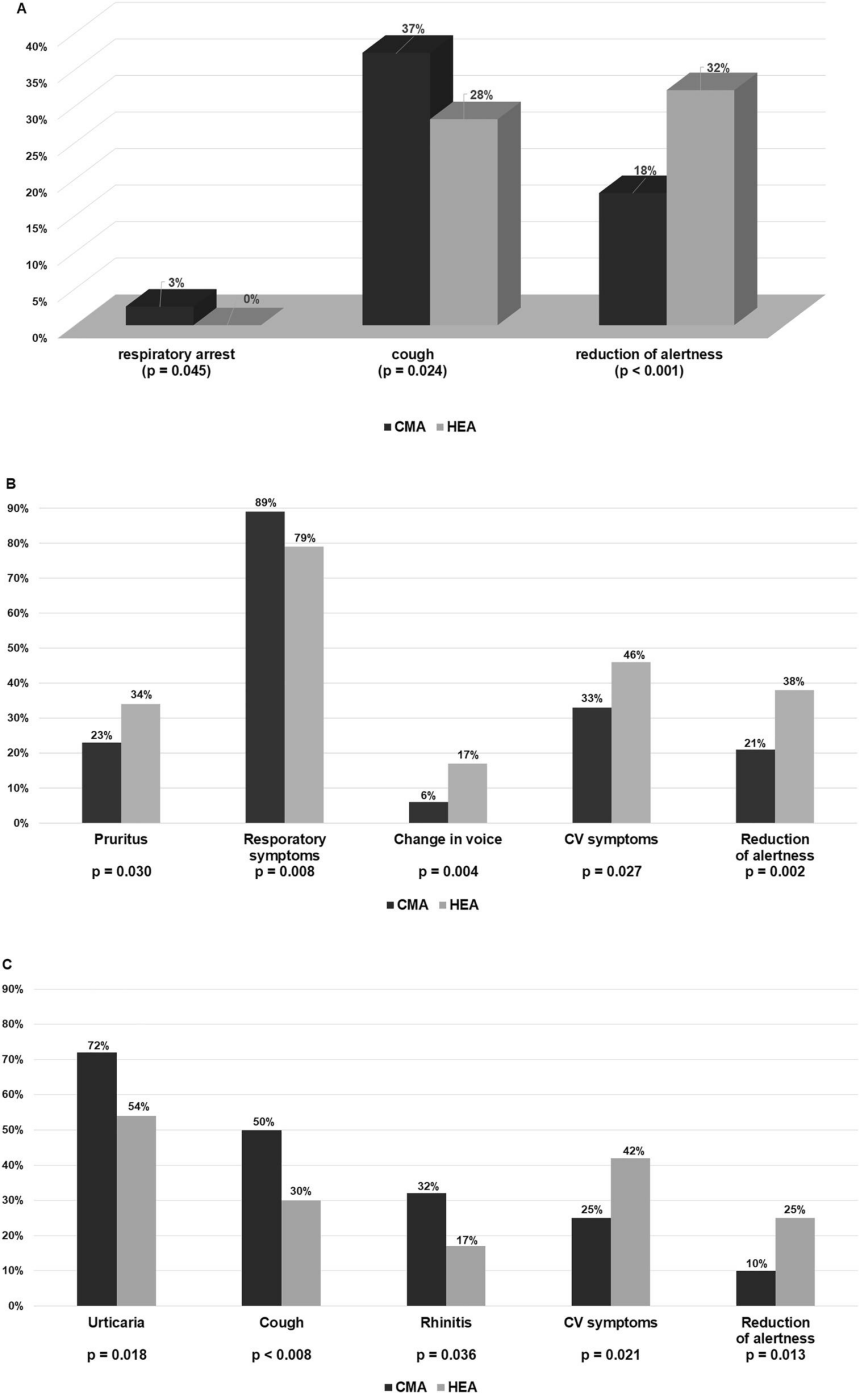
A, Significant differences between CMA versus HEA in clinical presentation. B, Significant differences in infants between CMA versus HEA in clinical presentation. C, Significant differences in children older than 1 year between CMA versus HEA in clinical presentation.

Skin symptoms, and then respiratory symptoms, were the most common signs of anaphylaxis, regardless of trigger. In detailed clinical presentation both triggers differed (Table S4). Respiratory symptoms occurred more often in the CMA children (*p* = 0.010), while cardiovascular symptoms were more common in the HEA patients (*p* = 0.002). Of the children with respiratory presentation, only rates of cough and respiratory arrest differed significantly between the groups (Figure 1A). Reduction of alertness predominated among the cardiovascular (CV) symptoms in both triggers (other CV symptoms did not exceed 7%), and occurred almost twice as often in the HEA sufferers.

In a separate analysis of infants and children older than 1 year, the two triggers differed from each other. In the infant group, CMA patients presented more frequently with respiratory symptoms in comparison to the HEA group. In contrast, pruritus, vocal changes, and CV symptoms, especially reduction of alertness, were more common in HEA infants (Figure 1B). In children older than 1 year, CMA patients more frequently presented with urticaria, cough, and rhinitis, while HEA children more often revealed CV symptoms, especially reduction of alertness (Figure 1C).

In cluster analysis, three clusters for each trigger group, separately, were identified. In the CMA children in the first cluster (40%) infants predominated, and the leading symptoms were erythema, flush and vomiting. In the second cluster (35%), mainly infants reacted, and mostly with angioedema and urticaria. In the third cluster (25%), the predominating symptoms were dyspnoea, cough, urticaria, and pruritus.

Similarly, in the HEA children, in the first cluster (40%) infants predominated, with vomiting and reduction of alertness as the leading symptoms. In the second cluster (16%), conjunctivitis was present in all children. In the third cluster (44%), neither conjunctivitis nor vomiting were reported, while nausea was more often observed than in the other clusters.

In over 50% of the children, the time from exposure to the allergen to the onset of symptoms in both trigger groups did not exceed 10 min, however this number was significantly higher in the CMA children compared to the HEA group (Table 1A). In the HEA children, more patients reacted after more than 30 min, both in the whole group and in the subgroup of repeated reactors (Table 1A,B). The rate of biphasic reactions was low, but was four times higher in the HEA children in comparison to CMA group (Table 1A).

R&M grade IV anaphylactic reaction was observed in eight children exclusively in the CMA group (88% males, 75% less than 1 year old, 38% from Brazil). In cases with a known reaction time, the episode occurred within 10 min of exposure. Approximately 50% of these children had previously reacted to CM. One case of death was reported, of a 9‐year‐old boy from France (Table 2).

**TABLE 2:** Clinical characteristics of children with grade IV CMA.

Country	Sex	Age	Fatal	Life threatening symptoms	Ever reacted	Amount of allergen	Atopy	Number of adrenaline injections
Brazil	M	4 months	No	Respiratory arrest	No	Unknown	Asthma + AD	1
Brazil	M	3 months	No	Respiratory arrest	No	Unknown	No	1
Brazil	M	1 year	No	Cardiac arrest	yes	Unknown	Asthma	Unknown
France	M	8 years	No	Wheezing, respiratory arrest, loss of consciousness, hypotension	yes	Half cup	Asthma	2
France	M	9 years	yes	Wheezing, respiratory arrest, cardiac arrest	yes	Unknown	No	Unknown
Germany	F	1 year	No	Respiratory arrest, loss of consciousness, hypotension	yes	Unknown	No	No
Switzerland	M	1 year	No	Respiratory arrest	No	Unknown	No	2
Switzerland	M	3 months	No	Respiratory arrest	No	1 cup	No	1

### 3.3 Pharmacological interventions and preventive measures

There was no significant difference between the CMA and HEA groups regarding the person (lay or professional) providing first‐line treatment (Table 3). The difference in terms of who provided the first‐line treatment concerned only the type of professional (Table 3). In first‐line, self‐administered treatment, antihistamines (AH1) were the most commonly used intervention, regardless of trigger. However, only glucocorticoids (GCS) were given 1.5 times more often in the CMA group compared to the HEA group. On the other hand, in first‐line professional intervention, the only difference manifested as the more frequent administration of AH1 to HEA sufferers, in comparison to the CMA sufferers The groups did not differ in terms of frequency of adrenaline administration both in lay as well as in professional intervention (Table 3).

**TABLE 3:** Detailed pharmacological intervention and preventive measures in CMA and HEA group.

	CMA		HEA		p
	n	%	n	%	
1^st^ line	238	88	178	92	NS
1^st^ line treatment by:					NS
No treatment	32	12	15	8	
Solely lay	59	22	45	23	
Solely professional	145	54	106	55	
Lay followed by professional	34	13	27	14	
**1** ^st^ **line professional treatment by:**					**0.002**
Allergy specialist	12	7	9	7	
Non‐allergy specialist	14	8	15	11	
Emergency doctor	96	55	56	42	
General practitioner	12	7	30	23	
Emergency healthcare professional	6	3	5	4	
Clinic or other person	35	20	18	14	
1^st^ line self‐administered treatment					
Adrenaline IM	24	26	10	14	NS
AH1	72	78	61	86	NS
**GCS**	**47**	**51**	**25**	**35**	**0.043**
SABA	25	27	13	18	NS
1^st^ line professional treatment					
Adrenaline	70	42	45	37	NS
**AH1**	**93**	**56**	**83**	**69**	**0.036**
GCS	116	70	79	65	NS
SABA	44	27	21	17	NS
2^nd^ line treatment	40	17	36	20	NS
Adrenaline	7	18	7	19	NS
AH1	28	74	25	69	NS
GCS	22	58	20	56	NS
SABA	7	18	5	14	NS
**Prescription of emergency drugs**	**240**	**87%**	**191**	**96**	**<0.001**
Adrenaline prescription	195	83	155	82%	NS
AH1 prescription	226	96%	183	97%	NS
**GCS prescription**	**204**	**87**	**140**	**74**	**0.001**
SABA prescription	78	33	47	25	NS
Counselling in avoidance	267	96	196	99	NS
Training in emergencies	256	92	188	95	NS

*Note*: Bold values indicate significant statistical differences.

Abbreviations: AH1, antihistamines; GCS, glucocorticosteroids; SABA, short acting β‐agonists.

**FIGURE 2:**
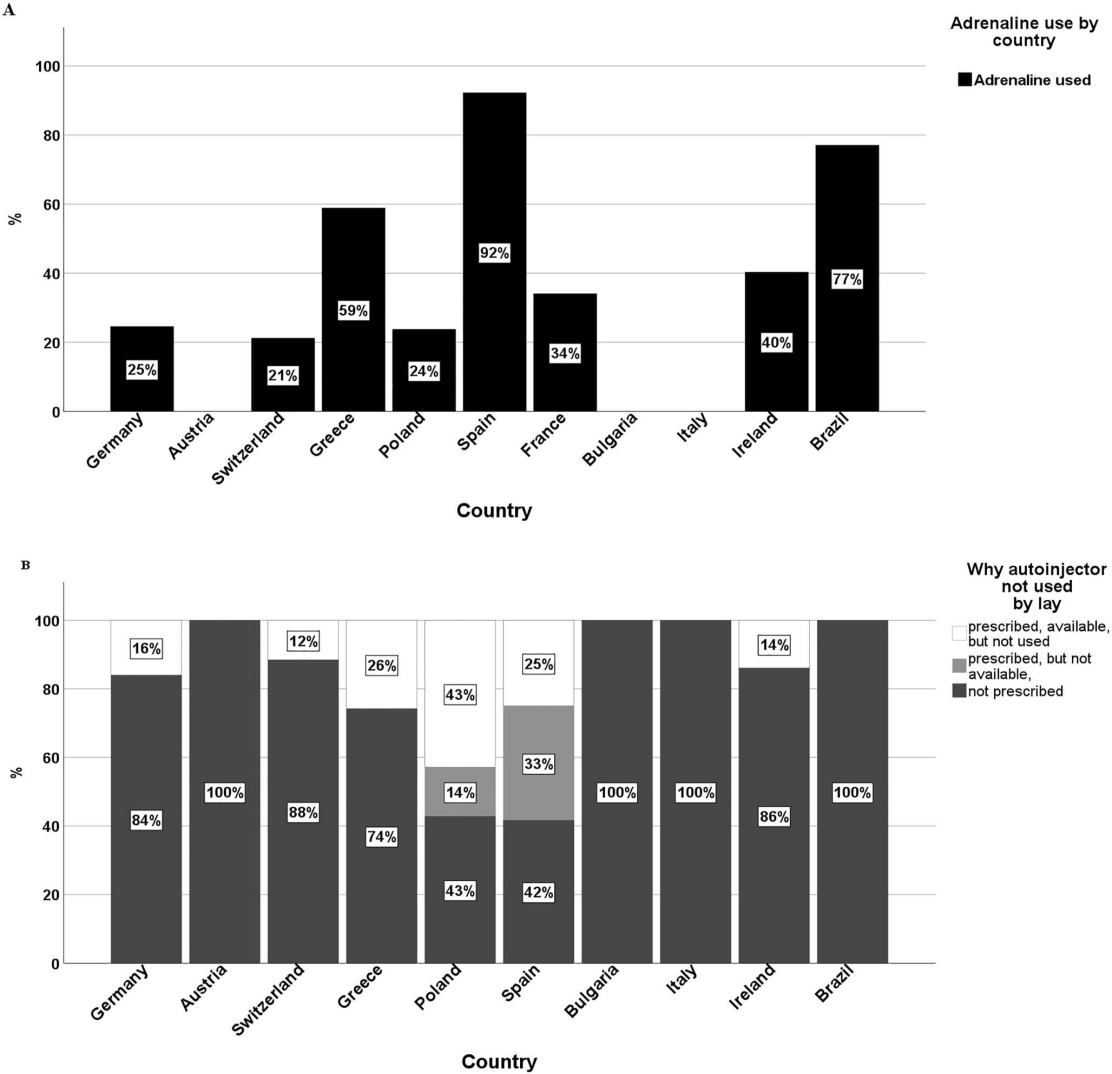
A, Adrenaline i.m. administration at the first‐line intervention in the countries. B, Reasons for the patient of not using adrenaline with respect to the countries.

The frequency of adrenaline intramuscular (i.m.) administration whether by medical staff or lay persons, in first‐line interventions, varied between countries (Figure 2A), as did patients' reasons for not using AAI (*p* < 0.001) (Figure 2B). In four countries, the only reported reason was the lack of a prescription for adrenaline. This reason was reported more frequently for children from the HEA group than the CMA group (74% compared to 86%, *p* = 0.017). In repeated reactors, a lack of prescription for adrenaline was reported as the reason for adrenaline not being used in 59% of CMA and 69% of HEA children, respectively.

About 50% of patients from both groups were hospitalized during an episode of anaphylaxis, without any difference in the rate of admission to their local Paediatric Intensive Care Unit (PICU), which did not exceed 5% (Table 1A).

The prescription of emergency drugs either prior the episode, in the emergency situation or during follow‐ups, was lower in CMA than in HEA. There was no difference in frequency of adrenaline prescription between the groups. At all stages of intervention in CMA children, in comparison to HEA, GCS were more frequently prescribed (Table 3).

According to the logistic regression model, the odds ratio (OR) regarding the use of adrenaline was higher for Brazil and Greece than for Germany, in both trigger groups. A similar observation in comparison to Germany was reported in children with CMA in Austria, Bulgaria, Italy, and Spain. In the CMA group, the OR of adrenaline use was higher in the children aged 4–6 years, compared to the infants. Similarly, the OR was higher in boys than in girls (Table 4).

**TABLE 4:** Model of multivariate analysis of the first dose of adrenaline injection.

	CMA				HEA			
	OR	95% CI		*p*	OR	95% CI		*p*
Germany	1				1			
Switzerland	1.77	0.55	5.64	0.337	0.56	0.11	2.96	0.496
Greece	3.94	1.30	11.96	**0.016**	5.78	1.59	20.95	**0.008**
Poland	0.19	0.02	1.94	0.160	1.84	0.44	7.74	0.407
France	2.00	0.62	6.44	0.245	1.28	0.37	4.41	0.696
Ireland	1.97	0.56	6.97	0.292	2.36	0.88	6.31	0.087
Brazil	12.62	4.14	38.47	**<0.001**	16.04	1.63	158.30	**0.018**
Other EU countries*	7.46	1.87	29.72	**0.004**	1.13	0.11	12.12	0.917
Allergy known	1.40	0.56	3.51	0.468	1.72	0.55	5.40	0.353
Up to 1 year	1				1			
From 2 to 3 years	0.81	0.28	2.36	0.700	0.59	0.18	1.99	0.395
From 4 to 6 years	5.87	1.72	20.09	**0.005**	0.83	0.24	2.89	0.765
More than 6 years	2.71	0.76	9.69	0.125	1.07	0.20	5.78	0.935
Sex (male)	3.48	1.60	7.55	**0.002**	1.12	0.52	2.45	0.769

*Note*: Bold values indicate significant statistical differences.

*Austria, Bulgaria, Italy, Spain.

## 4 DISCUSSION

### 4.1 Epidemiology and demography

Although CM and HE are common food allergens worldwide, especially in the population of young children,^1^, ^2^, ^3^, ^4^, ^5^, ^6^, ^7^, ^8^, ^10^, ^13^, ^14^, ^15^ there are no publications directly comparing their phenotypes. Our aim was to evaluate similarities and differences between these trigger groups. The topic is clinically relevant, as CM remains the most important trigger, responsible for up to 26% of fatalities due to food anaphylaxis in school‐aged children and adolescents in some European countries.^10^ Also in Canadian/US data, CM was one of the three most common identified triggers of fatal anaphylaxis in children admitted to ICU.^16^ In this study the only fatality was also reported in the CMA group, which is in line with the results from EAR dataset for children and adolescents, where five fatalities exclusively due to food allergy were reported, two of which were triggered by CM.^1^ No fatalities in children due to HEA have been reported in these paediatric publications.^1^, ^4^, ^7^, ^10^, ^13^, ^14^, ^15^ In this study predominance of infants and males was observed in both trigger groups. Age distribution with predominance of infants^4^, ^13^, ^14^, ^15^ and males (from 51% to 68%)^1^, ^4^, ^7^, ^10^, ^13^, ^14^, ^15^ up to school age was also reported in other studies.

Recurrent anaphylaxis in the CMA group presented in more than half of the patients, which is higher than the one third reported by the previous paediatric EAR study regarding children up to 12 years,^1^ and 1.5 times higher in comparison to the HEA group from this study. Retrospective data from Portugal indicates a similar frequency of recurrent anaphylaxis (41%),^13^ as opposed to data from a prospective French study, where the frequency was half as high (18%).^17^ Difference in the reported recurrency of anaphylaxis might be trigger‐ or patient‐dependent. The former refers to a high potency of allergen (e.g. peanuts), the second to the personal susceptibility due to comorbidities and cofactors. Pathophysiological role of cofactors is due to a decrease in the threshold for allergen tolerance.^18^, ^19^ In this study, cofactors, mainly physical exercise, differed between the triggers only in repeated reactors and accompanied up to one fourth of HEA reactions. In comparison to previous paediatric EAR data, physical exercise accompanied the anaphylaxis episode in about one fifth of children aged up to 12 years^1^

Little data refers to a correlation between the severity of reaction and amount of ingested allergen protein.^20^ We also did not find such a relationship, although the distribution of product volumes varied between triggers, which may be due to differences in allergen consistency. Regardless of specific allergen components, a product with a higher density might have a greater potential to cause a reaction, which is also supported by the results of studies on anaphylaxis to peanuts, where the most reactions were caused by the volume of the allergen up to one teaspoon.^11^ Further study is needed to examine to what extent differences of the clinical profiles are related to matrix and/or absorption effects and/or the allergen itself.

### 4.2 Clinical phenotype

Grade III R&M severity of reaction, most common in both trigger groups, turns out to be 1.5 times higher than in the paediatric EAR reports on all allergens, including food,^1^ but it is similar to EAR results dedicated to a specific allergen,^11^ while different to French study results, where R&M grade II predominated (54%) both in infants and pre‐schoolers.^7^

In detailed clinical presentation published by other authors, the frequency of the leading symptoms (skin 70%–99%; respiratory 48%–84%, except Swedish study, where they equalled 16%), followed by GI presentation (30%–49%)^1^, ^4^, ^7^, ^13^, ^14^, ^15^ occurring in the course of anaphylaxis, was similar to some extent to the results of this study. The fact that respiratory symptoms were more frequent in CMA, is probably due to higher prevalence of asthma in this trigger group. In Turkish infants respiratory symptoms and angioedema were the most frequent symptoms of food‐induced anaphylaxis in cases of tree nut–triggered symptoms, as compared to cow's milk and hen's egg (80.3%, 67.8%, and 64.5%, and 60.6%, 45%, and 45.2%, respectively).^15^ The greatest variation concerns the frequency of CV symptoms, which is reported within the range from 0% to 41%, increasing along with upper limit of cohort's age.^1^, ^4^, ^7^, ^13^, ^14^, ^15^ Such relationship may suggest that CV symptoms might be underdiagnosed, especially in younger children. In this study, CV presentation (especially reduction of alertness) was about 1.5 times more common in the HEA group, both in infants and older children, in comparison to CMA. The pathophysiology of such a phenomenon should be explored. Hypothetically, similarly to anaphylaxis to peanuts, such outcome may occur as a result of the insufficiency of compensatory mechanisms due to exposure to a strong potency allergen.^21^ Another hypothetical explanation concerns a similar way of acquiring food allergy to egg and peanuts by an impaired skin barrier, as both these allergens are strongly associated with the severity of atopic dermatitis.^22^

Additionally, we took an attempt to perform a more precise evaluation of clinical features for each allergen separately. For this reason, we used cluster analysis, which extracted three subgroups of cases homogenous in terms of clinical presentation for CMA and HEA, respectively. The clusters extracted in the CMA group may be characterised as: (1) mild dermal infants with severe GI, (2) severe dermal, (3) respiratory. While in HEA group: (1) infants with severe GI and/or reduction of alertness, (2) conjunctival, (3) mild GI without conjunctivitis. In both triggers groups in severe GI clusters, vomiting was a leading symptom, infants predominated, and both clusters constituted 40% of respective trigger group.

Cluster analysis is not frequently engaged in characteristics of study population in food anaphylaxis in children. In the first attempt of its use to explore the pattern of food anaphylaxis in children, two clusters were extracted.^23^ The first one, with severe anaphylaxis (R&M grade III‐IV), mainly due to nut allergy, and multiple atopic comorbidities, and the second one, described as mild anaphylaxis with various triggers and few atopic comorbidities.^23^ But, in French study CMA and HEA constituted only 8% and 7%, of the sample, respectively.^23^ Blazowski et al. in food systemic allergic reactions, identified two clusters – skin/non‐severe respiratory phenotype (81%) and severe respiratory/cardiovascular phenotype, but the study populations are not well comparable, as these authors graded severity by means of the 3‐stage scale by Muraro.^24^ All these mirror an attempts to characterize more precisely paediatric study population managed due to anaphylaxis.

Similarly to the previous EAR reports, where 58% of children reacted within 10 min of the exposure to allergen,^1^ data from this study shows that 67% of children with CMA and 53% of children with HEA developed symptoms within this period, including children with grade IV reactions. In Turkish infants the median time from allergen exposure to onset of symptoms was 10 min^15^ In Portuguese data, 88% of reactions started within 30 minutes of the exposure, regardless of trigger.^14^ The French paediatric study reported 26 min as the mean time between allergen exposure and the onset of symptoms.^7^ These differences may be due to the fact that French, Portuguese and Turkish data relates to all food allergens. Additionally, the difficulty of comparison is created by the differences in the way the results are presented. Biphasic reactions, though generally rare, were four times more common in the HEA group in comparison to CMA, being similar to the rate of 4.7% of biphasic reactions in the larger cohort from the EAR addressed to all triggers of anaphylaxis and all age groups,^25^ as well as to the previous paediatric data from EAR.^1^ Other data is sparse, a French and Turkish study included only one case of a biphasic reaction,^7^, ^15^ while the Portuguese reported none.^13^ Maybe the higher rate of biphasic reactions in HEA group is due to potential of allergen itself.

### 4.3 Pharmacological interventions and preventive measures

All international guidelines recommend adrenaline as the first line of intervention for anaphylaxis treatment,^26^ ^,^ ^27^ which is still underused, albeit with a tendency to improve in last few years.^28^ ^,^ ^29^ In this study, severity of grades III‐IV of anaphylaxis, being an absolute indication to adrenaline use, in total concerned about two thirds of children in each trigger group. About two thirds of these children received adrenaline in intervention. Underuse of adrenaline in anaphylaxis remains a universal problem, regardless of patients age, and geographical region and it is influenced by multiple factors.^28^ ^,^ ^30^ According to other European data, adrenaline in self‐administration ranges in children between 10% and 13%, while in professional intervention between 28% and 75%.^1^ ^,^ ^4^ What is more, our data showed regional differences (0%–92%) in AAI administration during the acute episode, the lowest rate being in Germany, and the highest in Brazil, Austria, Bulgaria, Italy, and Spain. This data is similar to the previous EAR reports, where the administration was significantly lower in German‐speaking countries (about 20%).^25^, ^28^ The problem should be considered in broader context, such as underuse of adrenaline due to under‐prescription, which variated from 42% to 100%, with the highest rate in Brazil, Italy, Bulgaria, and Austria. In repeated reactors, lack of adrenaline prescription was reported even as high as 59%–69% for CMA and HEA, respectively. This is similar to very high proportion (about 75%) of patients not prescribed AAI in children weighing below 15 kg with food‐induced anaphylaxis in large study from Japan.^31^ Given that the majority of severe food anaphylaxis events occur in children with a known food allergy, the supply of AAIs is of preventive importance. In other publication the most frequent reasons for not administering the AAI indicated by caregivers, included the following: reactions did not seem severe enough; it was the patient's first allergic reaction; use of other medication; and fear of using it.^30^ We did not observe any differences between the two anaphylaxis triggers regarding AAI prescription at any stage of intervention, though it concerned about 80% of patients. This result is slightly lower than the AAI prescription (90%) in EAR paediatric patients with food anaphylaxis.^1^

Low AAI prescription and refill rates have been recorded even in the absence of significant socio‐economic barriers, suggesting that economical limitations only partially account for the issue and cultural restrictions should also be considered.^32^ All these reflects the need for continued advocacy for the optimal management of food‐induced anaphylaxis and implementation of educational strategies regarding universal preventive measures (avoidance strategies, intervention drugs) and the primacy of adrenaline and the not primacy of GCS in treating anaphylaxis in children.^33^, ^34^

## 5 STRENGTHS AND LIMITATIONS

The strength of the study arises from the large amount of data derived from several countries, mostly European. However, the data was gathered from selected allergy clinics, and so it may be not representative of any one country as a whole. Another strength is the standardised manner of the data acquisition and the high quality of the data on the allergological assessments, gathered from the leading allergology centres in Europe and Brazil. Retrospective data collection could be considered a limitation of the study.

## 6 CONCLUSIONS

Despite the similarity of age, sex and severity grade, the clinical features and reaction profiles differed between CMA and HEA in children. Adrenaline was underused in treatment, while its administration was country dependent. Further studies are needed to examine to what extent differences in the clinical profiles are related to matrix and/or absorption effects, and/or the allergen itself.

## AUTHOR CONTRIBUTIONS

**Ewa Cichocka‐Jarosz**: Conceptualization (Equal); Formal analysis (lead); Investigation (Equal); Methodology (Equal); Resources (Equal); Visualization (lead); Writing ‐ original draft (lead); Writing ‐ review & editing (Equal). **Sabine Dölle‐Bierke**: Conceptualization (Equal); Data curation (Lead); Formal analysis (Equal); Investigation (Equal); Methodology (Equal); Resources (Equal); Software (Equal); Visualization (Equal); Writing ‐ original draft (Equal); Writing ‐ review & editing (Equal). **Urszula Jedynak‐Wąsowicz**: Investigation (Equal); Resources (Equal); Writing ‐ original draft (Equal); Writing ‐ review & editing (Equal). **Dominique Sabouraud‐Leclerc**: Investigation (Equal); Resources (Equal); Writing ‐ review & editing (Equal). **Alice Köhli**: Investigation (Equal); Resources (Equal); Writing ‐ review & editing (Equal). **Lars Lange**: Investigation (Equal); Resources (Equal); Writing ‐ review & editing (Equal). **Nikolaos G. Papadopoulos**: Investigation (Equal); Resources (Equal); Writing ‐ review & editing (Equal). **Jonathan Hourihane**: Investigation (Equal); Resources (Equal); Writing ‐ review & editing (Equal). **Katja Nemat**: Investigation (Equal); Resources (Equal); Writing ‐ review & editing (Equal). **Kathrin Scherer Hofmeier**: Investigation (Equal); Resources (Equal); Writing ‐ review & editing (Equal). **Stephanie Hompes**: Investigation (Equal); Resources (Equal); Writing ‐ review & editing (Equal). **Hagen Ott**: Investigation (Equal); Resources (Equal); Writing ‐ review & editing (Equal). **Lucila Lopes de Oliveira**: Investigation (Equal); Resources (Equal); Writing ‐ review & editing (Equal). **Thomas Spindler**: Investigation (Equal); Resources (Equal); Writing ‐ review & editing (Equal). **Christian Vogelberg**: Investigation (Equal); Resources (Equal); Writing ‐ review & editing (Equal). **Margitta Worm**: Conceptualization; Lead, Data curation (Equal); Formal analysis (Equal); Funding acquisition (lead); Investigation (lead); Methodology; Supporting, Project administration (lead); Resources (lead); Software (lead); Supervision (lead); Validation (lead); Writing ‐ original draft (Equal); Writing – review & editing (lead).

## CONFLICT OF INTEREST STATEMENT

ECJ serves as ALK paediatric advisory board, AK got personal fees (advisory board) from Allergopharma and Menarini AG, travel grant from Stallergenes, JO’BH receives research funding, speaker fees and consultancy fees from Aimmune Therapeutics, research funding and speaker fees from DBV Technologies, and research funding from Johnson&Johnson, Clemens von Pirquet Foundation, Temple St Hospital Foundation and City of Dublin Skin and Cancer Hospital Charity, KN was paid Nutricia/Danone and AimmuneTherapeutics in the past 3 years, other co‐authors declare no conflict of interest.

## Supporting information

Supporting Information S1

Supporting Information S2

Supporting Information S3

Supporting Information S4

## Data Availability

Anonymized data are available under OSF accession number: DOI 10.17605/OSF.IO/KCDW9.
