# Supplementary figures and images for: Cow's milk and hen's egg anaphylaxis: A comprehensive data analysis from the European Anaphylaxis Registry
Source: Clin Transl Allergy. 2023 Mar 26;13(3):e12228. doi: 10.1002/clt2.12228 (PMC10040951; doi:10.1002/clt2.12228)

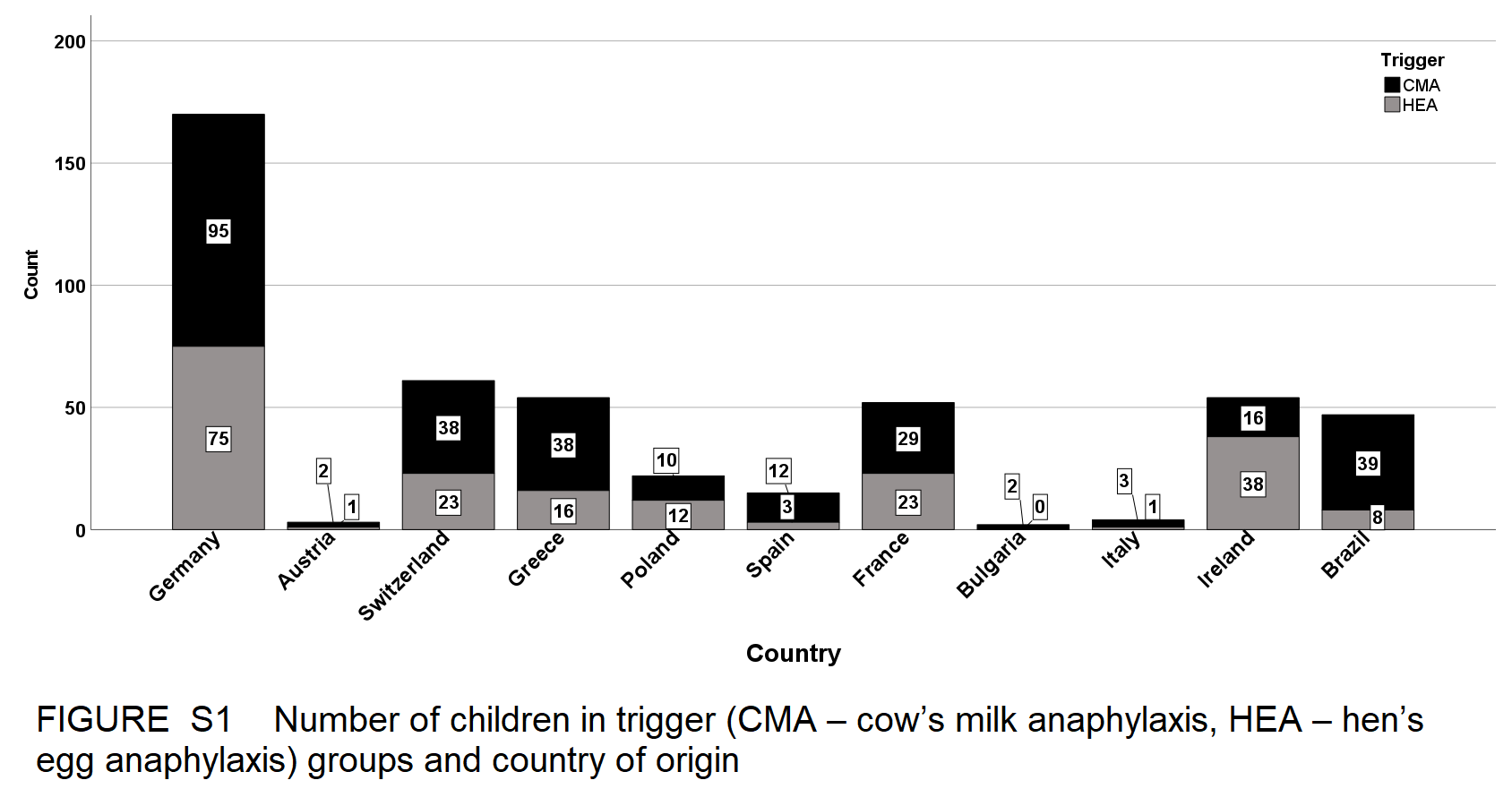

Supplement: Supplementary file 1 — Supporting Information S1 [file CLT2-13-e12228-s003.tif]

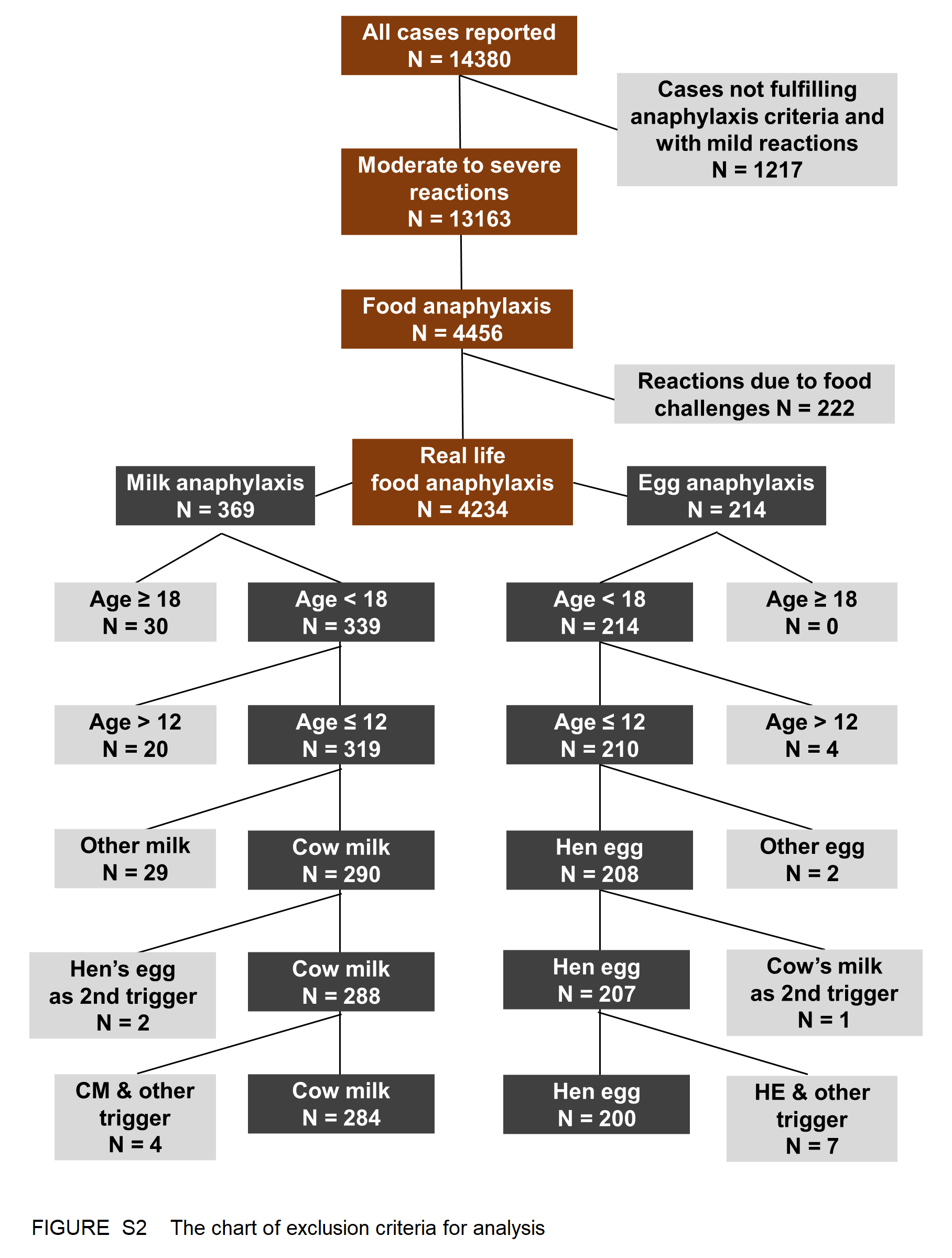

Supplement: Supplementary file 2 — Supporting Information S2 [file CLT2-13-e12228-s002.tif]

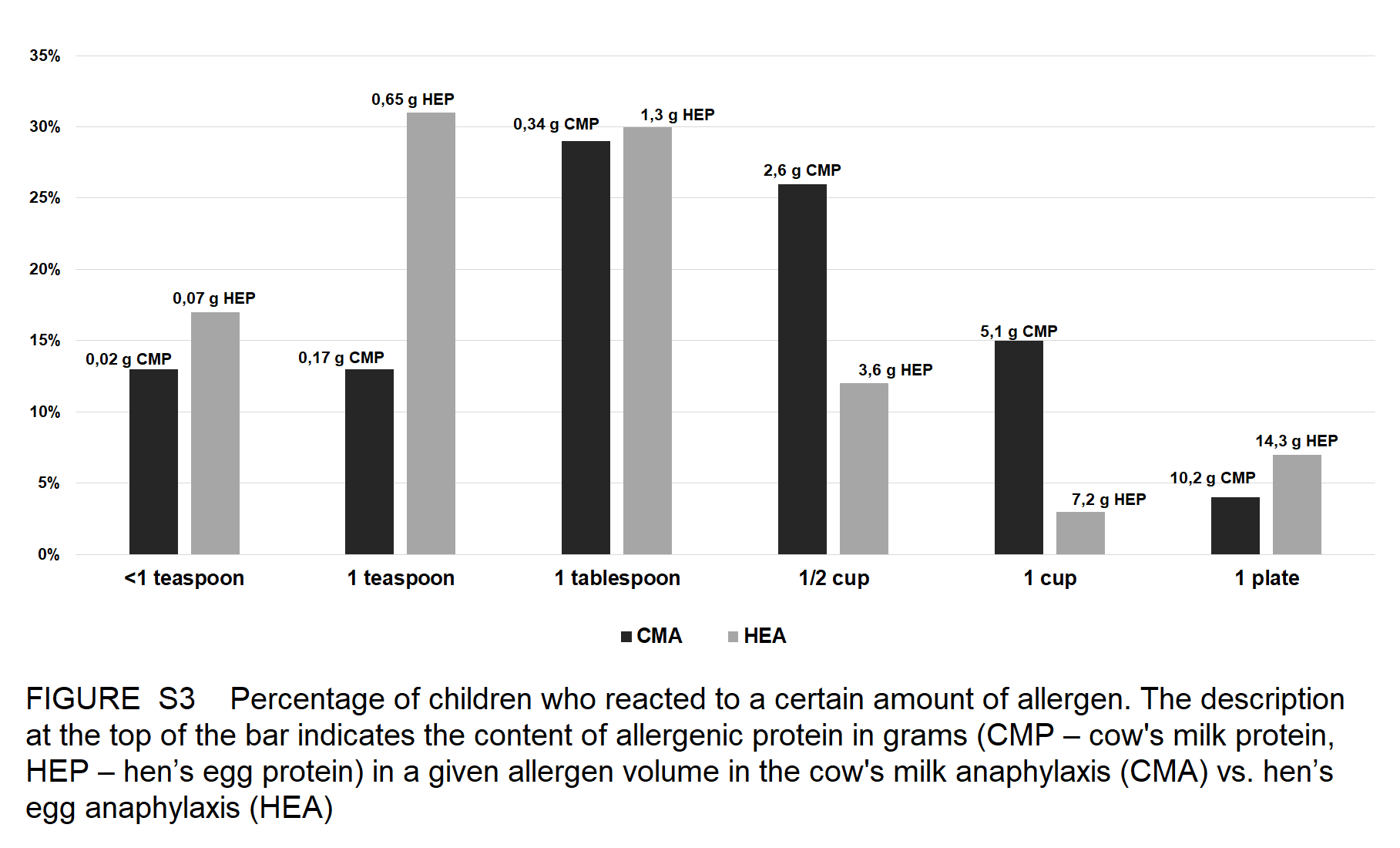

Supplement: Supplementary file 3 — Supporting Information S3 [file CLT2-13-e12228-s004.tif]
