# Supplementary material for: Cow's milk and hen's egg anaphylaxis: A comprehensive data analysis from the European Anaphylaxis Registry
Source: Clin Transl Allergy. 2023 Mar 26;13(3):e12228. doi: 10.1002/clt2.12228 (PMC10040951; doi:10.1002/clt2.12228)
Supplement: Supplementary file 4 — Supporting Information S4 [file CLT2-13-e12228-s001.docx]

TABLE S4 Detailed clinical presentation in CMA and HEA group

|  | Trigger | | | | p |
| --- | --- | --- | --- | --- | --- |
|  | CMA (n=284) | | HEA (n=200) | |  |
|  | N | % | N | % |  |
| **Skin** | **275** | **98** | **190** | **96** | **0,219** |
| Angioedema | 143 | 51 | 110 | 55 | 0,323 |
| Erythemaflush | 109 | 39 | 84 | 42 | 0,443 |
| Pruritus | 80 | 28 | 64 | 32 | 0,371 |
| Urticaria | 202 | 72 | 138 | 69 | 0,588 |
| Conjunctivitis | 27 | 10 | 31 | 16 | 0,061 |
| **GI** | **137** | **50** | **102** | **51** | **0,818** |
| Abdominal pain | 22 | 8 | 23 | 12 | 0,201 |
| Abdominal dist | 1 | 0 | 2 | 1 | 0,580 |
| Diarrhoea | 17 | 6 | 12 | 6 | 0,930 |
| Dysphagia | 3 | 1 | 7 | 4 | 0,107 |
| Vomiting | 122 | 45 | 77 | 39 | 0,193 |
| Incontinence | 0 | 0 | 2 | 1 | 0,177 |
| Nausea | 14 | 5 | 16 | 8 | 0,200 |
| **Respiratory** | **257** | **91** | **166** | **83** | **0,010** |
| Respiratory arrest | 7 | 2 | 0 | 0 | 0,045 |
| Dyspnea | 169 | 60 | 102 | 51 | 0,057 |
| Chest tightness | 5 | 2 | 3 | 2 | 1,000 |
| Cough | 97 | 37 | 52 | 27 | 0,024 |
| Change invoice | 23 | 9 | 27 | 15 | 0,071 |
| Throat tightness | 15 | 6 | 15 | 8 | 0,372 |
| Wheezing | 62 | 24 | 41 | 22 | 0,583 |
| Rhinitis | 57 | 22 | 35 | 18 | 0,349 |
| Stridor inspiratory | 28 | 10 | 28 | 14 | 0,165 |
| **CV** | **83** | **30** | **87** | **44** | **0,002** |
| Loss of consciousness | 12 | 4 | 6 | 3 | 0,456 |
| Hypotension collapsa | 18 | 7 | 10 | 5 | 0,478 |
| Chest pain angina | 0 | 0 | 0 | 0 | --- |
| Palpitation | 2 | 1 | 2 | 1 | 1,000 |
| Cardiac arrest | 2 | 1 | 0 | 0 | 0,512 |
| Dizziness | 11 | 4 | 9 | 5 | 0,769 |
| Tachycardia | 20 | 7 | 14 | 7 | 0,936 |
| Reduction of alertness | 48 | 18 | 64 | 32 | **< 0,001** |
| **Other symptoms** | **33** | **15** | **27** | **17** | **0,624** |
| Dysarthria | 0 | 0 | 0 | 0 | --- |
| Dysphonia | 1 | 0 | 1 | 1 | 1,000 |
| Hot sweat tremble | 8 | 4 | 5 | 3 | 0,805 |
| Paresthesia | 2 | 1 | 1 | 1 | 1,000 |
| Sight disorder | 0 | 0 | 0 | 0 | --- |
| Cyanosis pallor | 22 | 10 | 21 | 13 | 0,349 |
| Agony | 2 | 1 | 0 | 0 | 0,512 |
